# The Evolving Landscape of Immune Checkpoint Inhibitors and Antibody Drug Conjugates in the Treatment of Early-Stage Breast Cancer

**DOI:** 10.1093/oncolo/oyad233

**Published:** 2023-08-19

**Authors:** Prarthna V Bhardwaj, Yara G Abdou

**Affiliations:** Division of Hematology-Oncology, University of Massachusetts Chan School of Medicine-Baystate, Springfield, MA 01199, USA; Division of Oncology, University of North Carolina at Chapel Hill, Lineberger Comprehensive Cancer Center, Chapel Hill, NC 27514, USA

**Keywords:** breast cancer, immunotherapy, neoadjuvant therapy, adjuvant therapy, human epidermal growth factor receptor 2, immune checkpoint inhibitors, TROP2, antibody-drug conjugates, early-stage breast cancer, novel therapies

## Abstract

For decades, chemotherapy has been the mainstay of breast cancer treatment. Novel therapies are expanding the therapeutic options and altering the treatment algorithms to manage this disease. The use and approval of immune checkpoint inhibitors (ICIs) and antibody-drug conjugates (ADCs) represent a few areas of progress. These therapies initially gained attention in the metastatic setting but have subsequently found a role in early-stage breast cancer. Although human epidermal growth factor receptor 2 (HER2) is at the center of ADC development, other surface antigens with a differential expression between tumor and normal cells may be appropriate for ADC targeting. This has led to the discovery of new ADCs targeting other receptors, including TROP-2, HER-3, and LIV-1, to name a few. Similarly, the addition of pembrolizumab in treating early-stage triple-negative breast cancer has led to exploring other ICIs in this setting. However, it has also raised important scientific questions regarding optimal patient selection, biomarkers that predict the success of ICIs, ideal chemotherapy partners, and the financial implications of bringing newer therapies to the forefront. In this review, we discuss the evolving landscape of ICIs and ADCs in managing early-stage breast cancer and provide an overview of potential future advancement in the field.

## Teaser

This review reports on the use of immune checkpoint inhibitors and antibody-drug conjugates to manage early-stage breast cancer and provides an overview of potential future advancement in the field.

**Implications for Practice**

With strides made in discovering new molecular targets, there is a growing interest in exploring agents with novel mechanisms of action in the realm of breast cancer. These include immunotherapy and antibody-drug conjugates which are thought to be better tolerated than conventional cytotoxic chemotherapy and have also demonstrated improved outcomes. Early identification of optimal candidates for these therapies will help minimize toxicities and personify the true essence of personalized medicine.

## Introduction

**Figure 1.**
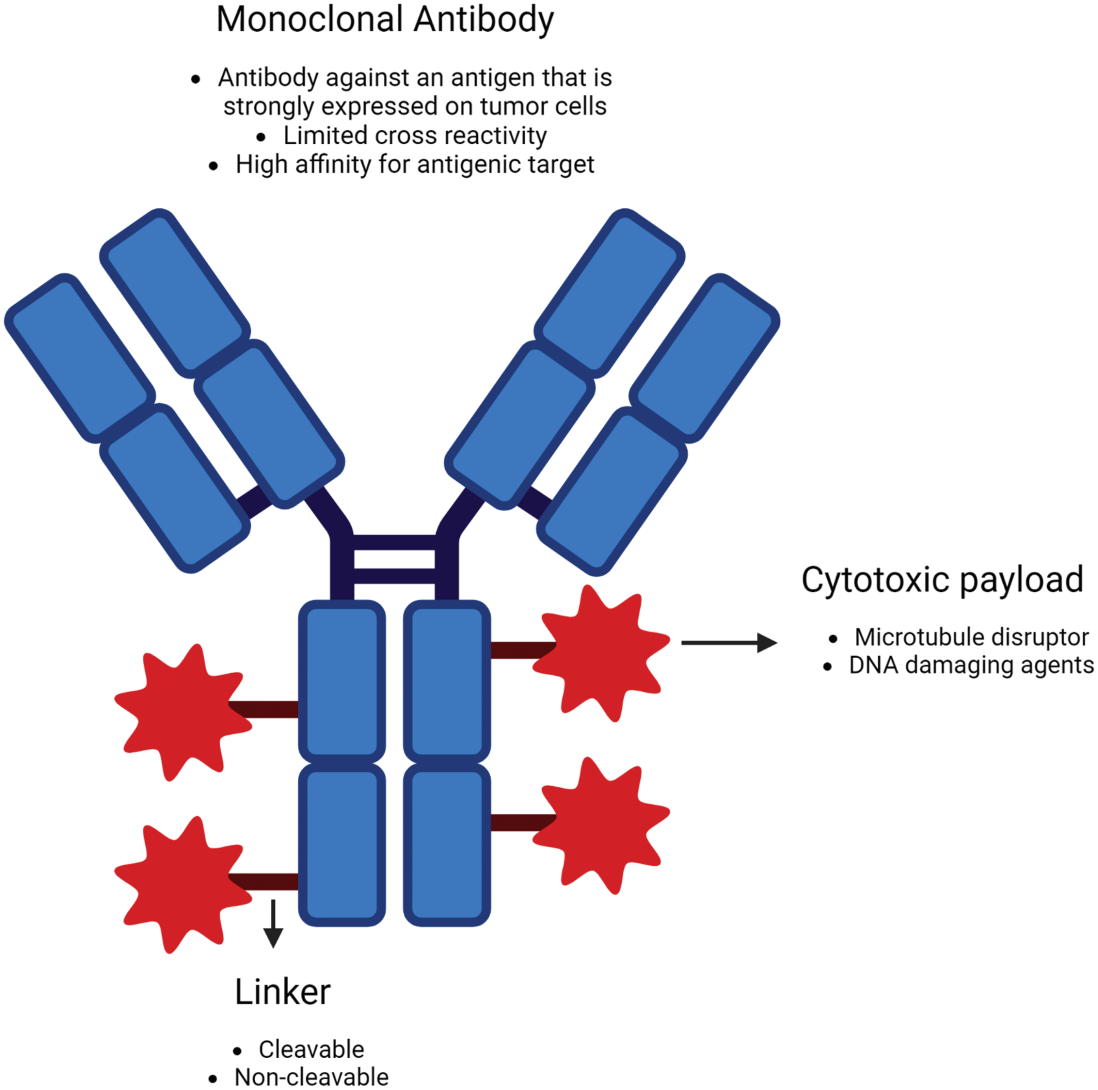
Antibody-drug conjugate structure. Created with BioRender.com

In the last few years, the development of innovative anti-cancer therapies with novel mechanisms of action has resulted in significant strides in the outcomes of patients with cancer, including breast cancer. Among these, immune checkpoint inhibitors (ICIs) and antibody drug-conjugates (ADCs) stand out. ADCs are designed to deliver potent cytotoxic agents that explicitly target cancer cells and spare normal cells, thus limiting toxicity while improving efficacy. ADCs comprise 3 key components, an antibody specific for a target antigen, a connecting linker, and a payload (Fig. 1). When the antibody binds to its specific antigenic target on the cancer cell surface, the ADC internalizes and processes to release the cytotoxic payload through lysosomal degradation, causing target cell death and, occasionally, neighboring cancer cells not expressing the antigen by a process called the “bystander effect.” Connecting linkers in ADCs may be cleavable or non-cleavable, triggering payload release based on factors like pH or enzyme-related changes.^1^ Due to the specificity conferred by the antigenic target, the cytotoxic payload of ADCs can be 100 to 1000 times more concentrated than is tolerated with traditional systemic chemotherapy.^2^

ICIs function by blocking critical immunosuppressive receptors such as programmed cell death (PD-1), programmed death-ligand 1 (PD-L1), and cytotoxic T-lymphocyte-associated protein 4 (CTLA-4).^3,4^ PD-1 is a vital inhibitory protein expressed on immune cells, including T cells, B cells, and antigen-presenting cells.^3^ When it binds to PD-L1, it induces apoptosis of antigen-specific T cells and decreases T-regulatory cell apoptosis, reducing the overall immune response. ICIs enhance immune surveillance and antitumoral response by blocking these regulatory proteins.

In this review, we analyze the landscape of ICIs and ADCs in managing patients with early-stage breast cancer, firstly discussing how their use came about and finally providing an overview of future directions in the field.

### Immunotherapy

Although once considered immunologically silent, expanding research in breast cancer has shed new light on the immunogenicity of this cancer. Expression of tumor-infiltrating lymphocytes (TILs) and PD-L1 differ based on subtypes of breast cancer, with triple-negative breast cancer (TNBC) displaying the highest expressions.^5-10^ These features are associated with an increased response to immunotherapy.^11^ Hence, ICIs were initially approved for treating patients with metastatic, PD-L1-positive TNBC based on improved survival outcomes.^12,13^ Evidence suggested a better efficacy of ICIs when administered early, in TNBC, potentially due to the progression of immune escape mechanisms during the disease advancement.^14,15^ Hence, treating patients with ICIs before surgery became a consideration.

#### Checkpoint Inhibitors in Triple-Negative Breast Cancer

The KEYNOTE-522 (KN522) brought ICIs to the forefront of treatment for patients with early-stage TNBC. This study demonstrated that the addition of pembrolizumab to chemotherapy preoperatively improved pathologic complete response (pCR) (63% vs. 55.6%, P < .001) and event-free survival (EFS) (84.5% vs. 76.8%, *P* < .001) compared to chemotherapy alone.^16^ Patients in the pembrolizumab arm received 9 cycles of adjuvant pembrolizumab after definitive surgery, regardless of pCR.

Several other studies have investigated the addition of ICIs to neoadjuvant chemotherapy in patients with early-stage breast cancer (Table 1). This includes the IMpassion031 trial, which improved pCR with the addition of atezolizumab to anthracycline-based chemotherapy (58% vs. 41%, *P* = .0044), especially in PD-L1-positive patients compared to PD-L1-negative patients (69% vs. 49%, *P* = .021), although no significant overall survival (OS) was noted.^19^ In contrast, the NeoTRIP study did not demonstrate a difference in pCR when patients received atezolizumab in combination with chemotherapy in the neoadjuvant setting (48.6% vs. 44.4%, *P* = .48).^21^ This outcome discrepancy could be attributed to a higher proportion of patients with locally advanced or stage III TNBC in NeoTRIP (~50% compared to 25% in other studies). Second, the choice of chemotherapy could also impact these results, where patients in NeoTRIP received an anthracycline-free backbone. This demonstrates that the role of the chemotherapy partner in the upfront setting remains crucial. Notably, atezolizumab for patients with early-stage TNBC has since been withdrawn in Europe based on the notion that the benefits of atezolizumab did not outweigh the risks in this population. However, it remains an option for patients in the rest of the world, subject to availability.

**Table 1.** Role of ICIs in in early-stage breast cancer.

Trial	KEYNOTE 522^16^	NeoPACT^28^	IMpassion031^19^	NeoTRIP^21^	GeparNuevo^17^	I-SPY2^43^
Design	Phase III	Phase II	Phase III	Phase III	Phase II	Phase II
Number of patients	1174	117	333	280	174	250
Disease setting	Stage II/III	Stage I—III	Stage II/III	Stage I-III (+N3)	Stage I-III	Stage II/III
ICI	Neoadjuvant/adjuvant pembrolizumab	Neoadjuvant pembrolizumab	Neoadjuvant/adjuvant atezolizumab for 1 year	Neoadjuvant atezolizumab 8 cycles	Neoadjuvant durvalumab 8 cycles	Neoadjuvant pembrolizumab 4 cycles
Chemotherapy backbone	Paclitaxel + carboplatin followed by AC	Carboplatin + docetaxel	*nab*-paclitaxel + AC	*nab*-paclitaxel/carboplatin + adjuvant anthracycline	*nab*-paclitaxel + epirubicin/cyclophosphamide	Paclitaxel + AC
pCR (ICI vs. placebo)	63% vs. 55.6% (estimated treatment difference, 7.5% (95% CI: 1.6%, 13.4%), P < .001)	60% (95% CI, 51%-70%)	57.6% vs. 41.1% (*P* = .0044)	48.6% vs. 44.4% (OR 1.18; 95% CI, 0.74-1.89, *P* = .48)	53.4% vs. 44.2% (OR 1.53; 95% CI, 0.82-2.84; *P* = .182)	60% vs. 22%
EFS/DFS/OS (ICI alone or vs. placebo)	EFS 84.5% vs. 76.8% (HR 0.63; 95% CI, 0.48-0.82, *P* < .001)	2-year EFS 88% in all patients	2-year EFS 80% vs. 83% (HR 0.76; 95% CI, 0.40-1.40) 2-year DFS 83 vs. 87% (HR 0.76; 95% CI, 0.44-1.30) 2-year OS 90% vs. 95% (HR 0.56; 95% CI, 0.30-1.04)	Pending	3-year iDFS 84.9% vs. 76.9% (HR 0.54; 95% CI, 0.27-1.09, *P* = .0559) 3-year OS 95.1% vs. 83.1% (HR 0.26, 95% CI, 0.09-0.79, *P* = .0076)	Numerically higher with small sample size and not powered for statistical significance

Abbreviations: AC: doxorubicin/cyclophosphamide; ICI: immune checkpoint inhibitors; pCR: pathologic complete response; OR: odds ratio; CI: confidence interval; EFS: event-free survival; DFS: disease-free survival; OS: overall survival; iDFS: invasive disease-free survival; HR: hazard ratio.

Furthermore, the GeparNuevo study evaluated the role of durvalumab in combination with chemotherapy in the neoadjuvant setting. Patients who received durvalumab versus placebo showed an improvement in 3-year disease-free survival (DFS) (85.6% vs. 77.2%, *P* = .036) but no statistical differences in pCR rates.^17^ A multivariable analysis demonstrated a durvalumab effect independent of pCR, suggesting that pCR may not be the sole benefit driver with ICIs. Also, a unique aspect of this study was the “window of opportunity” cohort (*n* = 117), where patients received a single induction dose of durvalumab or placebo prior to the commencement of the neoadjuvant treatment. A significant pCR benefit was seen in this cohort among patients who received durvalumab versus placebo (61.0% vs. 41.4%, *P* = .035). Although the cohorts were small, an increase in TILs was observed among durvalumab responders suggesting an activation of the immune system during the induction phase in these patients.

##### Challenges and Future Considerations

While there has been tremendous progress in integrating immunotherapy in the treatment of early-stage breast TNBC, several questions remain regarding the ideal patient selection, biomarkers predictive of response, optimal chemotherapy partners, and the role of adjuvant therapy.

##### Biomarkers Predicting Response

Biomarkers for predicting responses to ICIs can pave the way for a more personalized treatment approach. PD-L1, tumor mutational burden (TMB), and TILs are among the most widely explored predictive biomarkers for immunotherapy. PD-L1 is one of the first biomarkers investigated to predict response among patients with various cancers.^18^ In patients with breast cancer, while PD-L1 expression predicted response to pembrolizumab in the metastatic setting, improved responses to ICIs were seen regardless of PD-L1 status in the early-stage setting^12,19,20^; therefore, its value in predicting benefit to ICIs in patients with non-metastatic breast cancer is limited. Furthermore, patients with high-TMB have also demonstrated improved efficacy with immunotherapy, secondary to an increased ability of the tumor to produce neoantigens, making the tumor more immunogenic. This has been well demonstrated in breast cancer, specifically in patients with TNBC.^11,22,23^ The prognostic significance of TILs and their role in predicting response to neoadjuvant systemic therapy is evident in early TNBC.^24-27^ However, their role in predicting response to ICIs is still not well defined. The NeoPACT trial has shown that immune enrichment identified by stromal TILs was associated with higher pCR rates approaching or exceeding 80%.^28^ Moreover, the GeparNuevo study showed that stromal TILs independently predicted pCR rates in the ICI arm.^29^ Both these findings suggest that TILs may indeed be a good predictor of response. Further studies are needed to validate these biomarkers independently and prospectively as predictors of response to immunotherapy, especially in patients with early-stage breast cancer. Work is underway to identify signatures that better identify patients with immunotherapy-responsive disease, including the 27-gene TME assay and ImPRINT assay.^30,31^

##### Role of the Chemotherapy Partner

An additional conundrum is the optimal chemotherapy partner that can aid in maximizing the benefit from ICIs for patients with early-stage breast cancer. Most ICI trials utilized an anthracycline and taxane-based chemotherapy backbone. Although, NeoPACT, a single-arm phase II study where patients received an anthracycline-free regimen (carboplatin and docetaxel) in combination with pembrolizumab in the preoperative setting, resulted in 60% pCR rates with a 2-year EFS of 88%.^28^ This is comparable to pCR from studies utilizing anthracyclines in combination with ICIs. In contrast, the NeoTRIP study, which exclusively had patients receiving carboplatin and *nab*-paclitaxel with atezolizumab preoperatively and allowed the use of anthracyclines at the investigator’s discretion in the post-neoadjuvant setting, demonstrated no differences in pCR (48.6% vs. 44.4%, *P* = .48).^21^ Therefore, it is currently unclear if we can omit anthracyclines in the upfront setting. To address this further, the proposed SWOG 2212 trial (SCARLET) will evaluate EFS among patients receiving an anthracycline (KN522 regimen) versus a non-anthracycline (NeoPACT regimen) chemotherapy backbone. This is particularly important among older adults, who are often not considered ideal candidates for anthracycline use.

##### Optimal Adjuvant Treatment

Based on KN522, the current standard of care is for patients to receive adjuvant pembrolizumab post-neoadjuvant therapy and definitive surgery, regardless of pCR. Other studies, like GeparNuevo, did not incorporate ICIs for patients in the adjuvant setting, yet demonstrated an improvement in EFS.^17^ Therefore, the role of ICIs in the adjuvant setting is yet to be defined, particularly where pCR is achieved. To study this further, the proposed OptimICE-pCR trial will compare survival outcomes with and without adjuvant pembrolizumab in TNBC patients who have received neoadjuvant therapy with chemoimmunotherapy and achieved pCR.^32,33^

Likewise, the optimal adjuvant therapy in patients with TNBC and residual invasive disease remains undefined. Capecitabine was first approved in patients with residual invasive disease following definitive surgery based on the results of the CREATE-X trial, which demonstrated a superior OS with adjuvant capecitabine in the TNBC subgroup (78.8% vs. 70.3%, HR 0.52; 95% CI, 0.30 to 0.90).^34^ Subsequently, olaparib was approved based on the OlympiA study in patients with high-risk human epidermal growth factor receptor (HER2)-negative breast cancer, harboring a germline BRCA mutation and residual invasive disease following neoadjuvant therapy and surgery or node-positive disease following upfront surgery requiring adjuvant chemotherapy. The study demonstrated an improved 4-year invasive DFS (82.7% vs. 75.4%; difference 7.3%, 95% CI, 3.0% to 11.5%) with adjuvant olaparib for 1 year.^35^ Notably, KN522 did not incorporate either of these therapies with pembrolizumab in the adjuvant setting. Hence, the best and safest way of integrating immunotherapy with prior standard-of-care treatments is yet to be evaluated in clinical trials. Based on expert consensus and safety data available from the use of these combinations in the metastatic setting, we may use pembrolizumab with capecitabine in patients with residual invasive disease and pembrolizumab with olaparib for high-risk BRCA-mutant patients with residual disease.^36^ To address this further, ASCENT-05, an ongoing multi-arm phase III trial [NCT05633654], is assessing survival outcomes of adjuvant pembrolizumab monotherapy versus combination of pembrolizumab and capecitabine versus combination of pembrolizumab and sacituzumab govitecan (SG) in patients with residual disease following neoadjuvant therapy.

##### Toxicities, Accessibility, and Cost

Finally, the flip side of improving patient outcomes with ICIs is drug-related toxicities beyond those caused by cytotoxic agents, such as immune-mediated adverse effects. In addition, the negative impact of immunotherapy on fertility remains a genuine concern in this subset of patients who are traditionally younger and premenopausal.^37^ Besides conventional toxicities, the use of ICIs also adds to financial toxicity to the patient and the healthcare system with a resultant potential for disparities in care. Therefore, ongoing studies to identify the ideal candidates for ICIs and optimize the duration of use could help minimize these effects.

#### Checkpoint Inhibitors in Other Types of Breast Cancers

Various early-phase trials have studied the combination of ICIs and anti-HER2 monoclonal antibodies in patients with metastatic HER2-positive breast cancer, particularly in PD-L1-positive disease.^38-40^ In the early setting, IMpassion050, a phase III study, where patients received neoadjuvant chemotherapy and anti-HER2-directed antibodies with or without atezolizumab, demonstrated no difference in pCR rates (62.7% vs. 66.1%, *P* = .9551).^41^ There are other ongoing phase III trials, including APTneo [NCT03595592] and ASTEFANIA [NCT04873362], which are evaluating EFS and invasive DFS, respectively, using ICIs in combination with ADCs in patients with early-stage HER2-positive breast cancer.

ICIs are also being studied in patients with hormone receptor-positive (HR+), HER2-negative breast cancer. Although response rates in the metastatic setting were low,^42^ findings from the randomized phase II I-SPY 2 trial (NCT01042379) showed that adding an ICI to neoadjuvant chemotherapy in the early-setting can improve pCR rates.^43^ KEYNOTE-756 [NCT03725059] and CHECKMATE 7FL [NCT04109066] are ongoing phase III trials evaluating ICI use in the early setting.

#### Novel Immunotherapies

**Figure 2.**
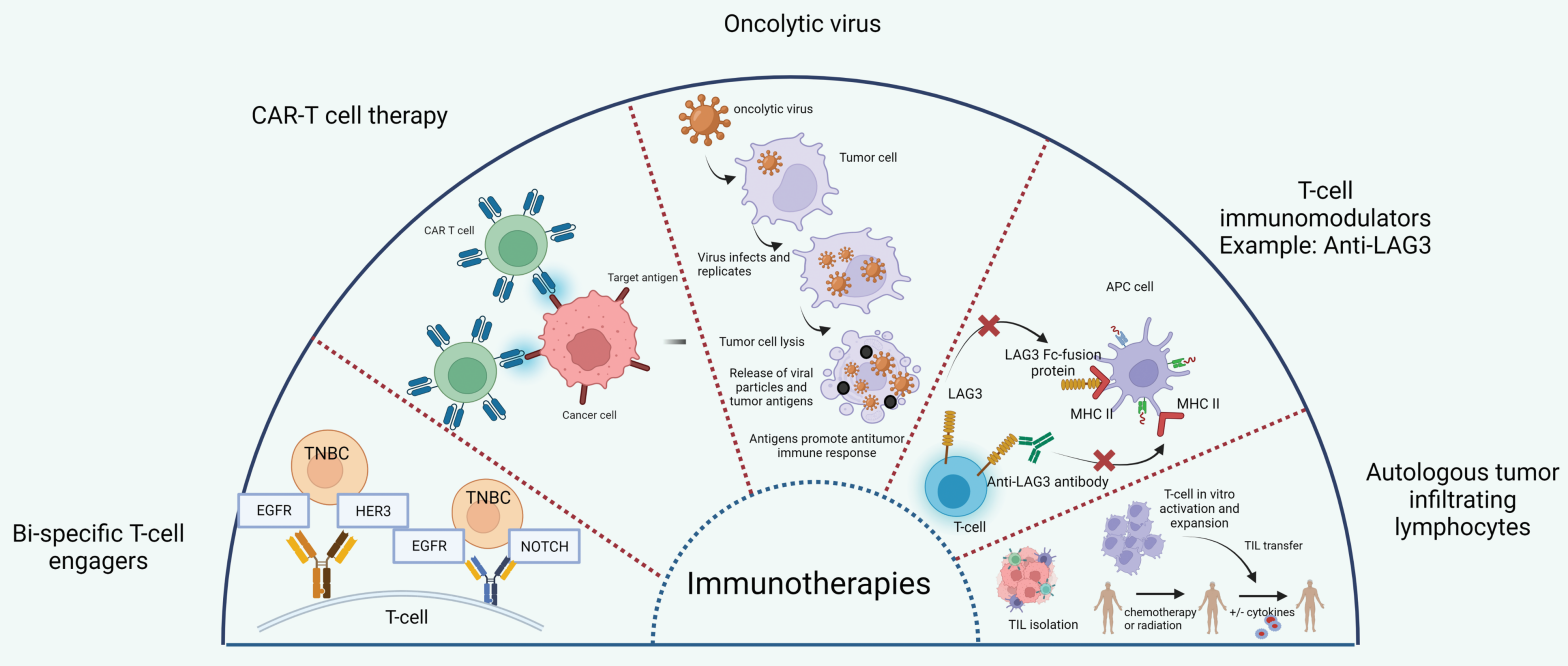
Novel immunotherapies with their mechanism of action. Created with BioRender.com

Various novel modalities of immunotherapy, including CAR-T cell therapy, bispecific T-cell engagers, and other immunomodulatory receptors like LAG-3 have been evaluated in patients with breast cancer, particularly in the metastatic setting^44^ (Fig. 2). Oncolytic virus therapy is one of the few modalities that has shown promising therapeutic efficacy in early-stage breast cancer. In a phase II clinical trial, patients with early-stage TNBC received intratumoral Talimogene-laherparepvec (T-VEC), an oncolytic virus, in addition to neoadjuvant chemotherapy. The study met its primary endpoint of residual cancer burden index (RCB) of 0 (corresponding to pCR) in 45.9% of patients, while 65% had RCB-I.^45^ Hence, this approach is touted as a novel way of enhancing immune activation, warranting further studies in this realm.

### Antibody-Drug Conjugates

**Figure 3.**
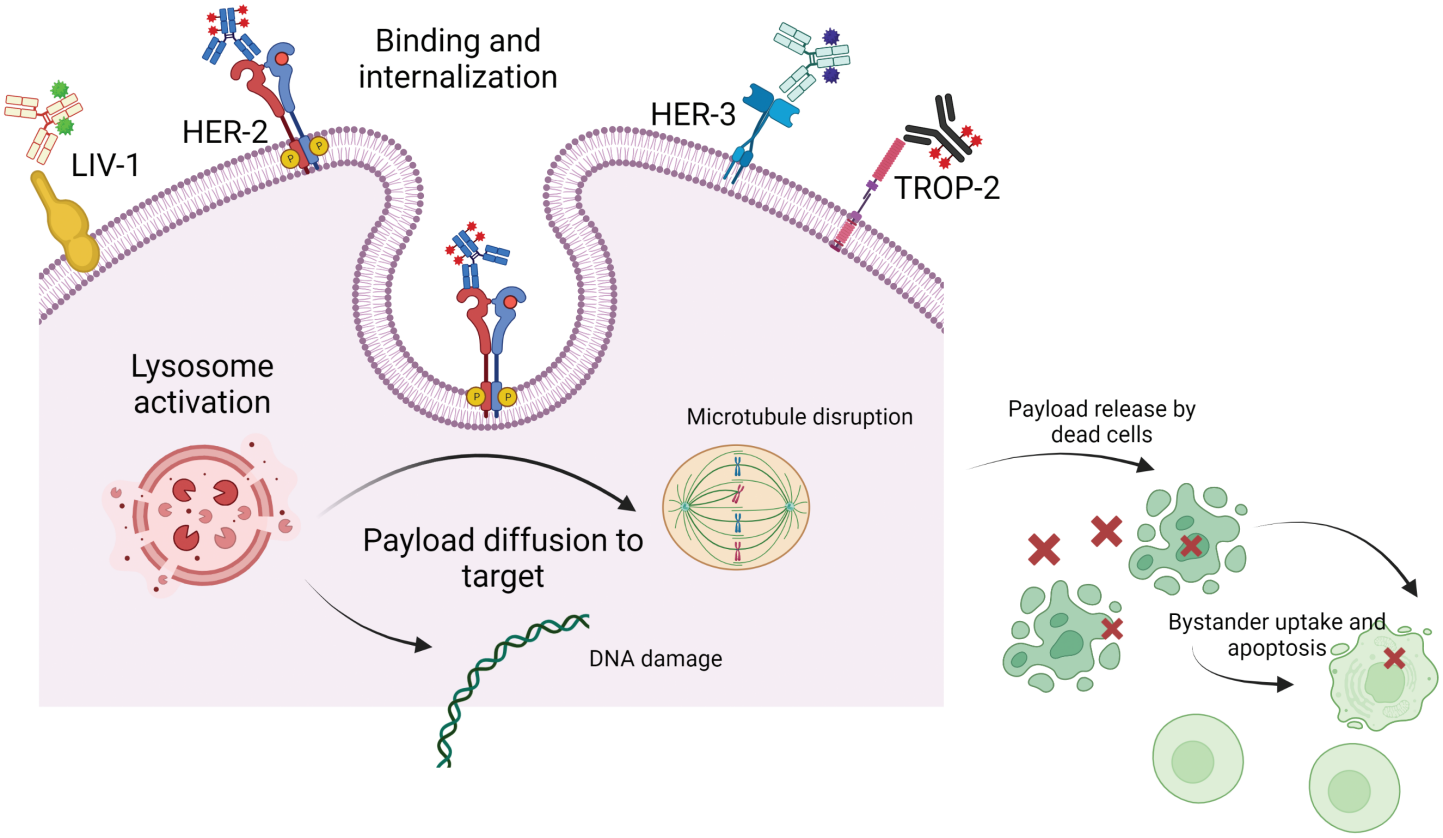
Mechanism of action of ADCs. Created with BioRender.com

ADCs are rapidly evolving therapies that can deliver potent chemotherapy specifically to cancer cells while largely sparing normal cells. Advances in antibody, linker, and payload technology have allowed the development of several classes of ADCs tailored for varying indications. Although HER2 led the way for the development of ADCs in breast cancer, other antigens such as TROP-2, HER-3, and LIV-1 have recently emerged as novel targets for ADC and represent a step forward (Fig. 3).

#### Human Epidermal Growth Factor Receptor-2 Antibody-Drug Conjugates

HER2 (also known as ERBB2) is a transmembrane glycoprotein that is part of the epidermal growth factor receptor (EGFR) family of receptors.^46^ HER2 dimerization triggers many cell signal pathways leading to cell proliferation and tumorigenesis.^46^ HER2 overexpression and amplification are also associated with a higher risk of disease recurrence and death.^47-49^ Breast cancers with HER2 expression became an attractive therapeutic target following the success of monoclonal antibodies over the years.^50,51^ ADCs against HER2, which can provide more potent cytotoxic therapies to cancer cells while limiting toxicity to surrounding normal cells, represent a promising option of this era.

##### Ado-Trastuzumab Emtansine

Ado-Trastuzumab Emtansine (T-DM1), the first ADC against HER2, links trastuzumab with a maytansinoid derivative DM1, an antitubulin that is ~24 to 270 times more potent than paclitaxel.^52^ It was initially approved for patients in the metastatic setting based on the results of the TH3RESA and EMILIA studies demonstrating improved survival outcomes in the third-line^53,54^ and second-line setting, respectively.^55,56^ Subsequently, the KATHERINE study catapulted T-DM1 to the adjuvant setting, where patients with residual invasive disease following neoadjuvant therapy with a taxane and trastuzumab, had a better 3-year invasive DFS (88.3% vs. 77.0%, *P* < .001) and lower risk of distant recurrence (89.7% vs. 83%) with 1 year of adjuvant T-DM1 compared to trastuzumab.^57^ However, there was no OS benefit noted to date with this approach.

Other completed and ongoing studies of T-DM1 in the adjuvant setting have been described in Table 2. Of importance, the ATEMPT trial, which randomized patients with stage I HER2-positive breast cancer to T-DM1 versus paclitaxel/trastuzumab (TH), was designed to determine if treatment with T-DM1 was less toxic than TH. The study showed excellent efficacy with T-DM1 (3-year iDFS of 97.8%) and similar incidence of clinically relevant toxicities among patients in both arms. However, differences in adverse event profiles were noted, where T-DM1 was associated with less neuropathy and alopecia, and improved work productivity among patients. Therefore, it is essential to weigh the patient’s preferences while choosing the ideal drug in this setting.^61^

**Table 2.** T-DM1 in the neoadjuvant/adjuvant setting.

Trial	Design	Setting	Treatment	Main outcomes
Neoadjuvant studies completed				
ADAPT^58^	Phase II umbrella trial *N* = 5000	Triple-positive (ER/PR/HER2) early breast cancer	Neoadjuvant trastuzumab plus endocrine therapy (control) or T-DM1 or T-DM1 plus endocrine therapy	pCR: 15.1% vs. 41% vs. 41.5% (*P* < .001)
I-SPY 2^59^	Adaptive phase II trial *N* = 248	HER2-positive breast cancer larger than 2.5 cm	Neoadjuvant paclitaxel plus trastuzumab or T-DM1 plus pertuzumab followed by preoperative doxorubicin plus cyclophosphamide	pCR: 22% vs. 52% In HR + patients, pCR was 17% vs. 46% In HR patients, pCR was 33% vs. 64%
KRISTINE ^60^	Phase III N = 444	HER2-positive stage II or III operable breast cancer	Neoadjuvant T-DM1 plus pertuzumab or docetaxel, carboplatin, and trastuzumab plus pertuzumab	pCR: 44% vs. 57%
Adjuvant studies completed				
ATEMPT^61^	Phase II *N* = 500	Stage I HER2 positive breast cancer	One year of adjuvant T-DM1 vs. 12 weeks of trastuzumab plus paclitaxel	Clinically relevant toxicity: 46% vs. 47% (*P* = .83) 3-year DFS in T-DM1: 97.8% (exceeding prespecified 3-year DFS of 95%)
Ongoing studies				
CompassHER2 RD [NCT04457596]	Phase III *N* = 1031	HER2 positive cT1-T4, N0-3 preoperatively with residual disease	One year of adjuvant T-DM1/placebo vs. T-DM1/tucatinib	Primary outcome: iDFS Secondary outcome: distant RFS, OS
ASTEFANIA [NCT04873362]	Phase III *N* = 1700	Stage I-III HER2-positive breast cancer treated with neoadjuvant therapy and residual disease	One year of adjuvant T-DM1/placebo vs. T-DM1/atezolizumab	Primary outcome: iDFS Secondary outcome: DRFI, OS

Abbreviations: ER: estrogen receptor; PR: progesterone receptor; HER2: human epidermal receptor; T-DM1: ado-trastuzumab emtansine; pCR: pathologic complete response; HR: hormone receptor; DFS: disease-free survival; iDFS: invasive disease-free survival; RFS: recurrence-free survival; DRFI: disease recurrence free interval; OS: overall survival.

Two other ongoing large phase III trials, CompassHER2 RD and ASTEFANIA, are evaluating the role of adjuvant T-DM1 in combination with tucatinib and ICI, respectively, for patients with residual disease post-neoadjuvant therapy.

##### Trastuzumab Deruxtecan

Trastuzumab Deruxtecan (T-DXd), the second ADC approved for the treatment of advanced HER2-positive breast cancer, is unique compared to T-DM1 in several ways. First, T-DXd has a cleavable linker with a membrane-permeable payload that exhibits the “bystander effect."^62,63^ This is in contrast to T-DM1, which has a non-cleavable linker that is dependent on lysosomal degradation and travels to the cytoplasm for the cytotoxic payload to exhibit its effects. In addition, the payload in T-DXd is an exatecan-derived topoisomerase-I inhibitor, in contrast to maytansine DM1 in T-DM1. Finally, T-DXd has a higher drug-antibody ratio of 8 compared to T-DM1’s ratio of 3.5. These differences can possibly explain the efficacy seen with T-DXd in HER2 low disease and disease that is refractory to T-DM1.^62,64^

The DESTINY-Breast trials have demonstrated the impact of T-DXd in various settings. T-DXd was first approved in the metastatic setting based on the phase II DESTINY-Breast 01 trial, where patients had an overall response rate of 60.3% and a median PFS of 16.4 months.^65^ This translated into an ongoing survival benefit for patients in the phase III DESTINY-Breast 02 trial, the first randomized study to show the benefit of an ADC post another ADC.^66^ The DESTINY-Breast 03 trial then directly compared T-DXd with T-DM1 in metastatic HER2-positive breast cancer, showing the longest reported median PFS among patients with HER2-positive breast cancer (28.8 months vs. 6.8 months, *P* < .0001) and a significant improvement in OS with T-DXd (not reached).^67^ Based on these remarkable survival outcomes, T-DXd is being extensively studied in patients with early-stage disease. In particular, DESTINY-Breast 05 is a phase III trial directly comparing adjuvant T-DXd with T-DM1 in patients with residual disease following standard neoadjuvant therapy. Furthermore, DESTINY-Breast 11 will be the first trial to evaluate T-DXd in the neoadjuvant setting in patients with high-risk HER2-positive early-stage breast cancer.

An intriguing feature of T-DXd has been its efficacy not only in patients with HER2-positive disease but also in HER2 low breast cancer, which is defined as 1+ by immunohistochemistry (IHC) or 2+ by IHC and negative by Fluorescence in situ hybridization (FISH). Based on pathologic HER2 classification, ~40%-60% of patients with breast cancer could meet the current criteria for HER2 low status, out of which nearly 80% are HR+ and 20% are HR negative.^68-70^ The DESTINY-Breast 04 trial demonstrated that patients with previously treated, metastatic HER2 low breast cancer had an improved PFS (9.9 vs. 5.1 months, *P* < .001) and OS (23.4 vs. 16.8 months, *P* = .001) with T-DXd compared to physician’s choice of chemotherapy.^71^ This represents a paradigm shift for this group of patients, who were otherwise ineligible for HER2-directed therapies. Other studies have evaluated the role of T-DXd in patients with HER2 low, HR+ early-stage breast cancer. The TALENT study is one such phase II clinical trial where patients received 6 cycles of T-DXd, with or without an aromatase inhibitor, in the neoadjuvant setting before surgery. Preliminary data demonstrated an overall response rate of 63% and 75% with or without an aromatase inhibitor, respectively.^72^ The results are intriguing, although further data are needed to validate these findings.

In summary, the unique characteristics of this ADC have made it an exciting therapeutic option for patients with HER2-positive and HER2 low breast cancer. However, T-DXd is not currently standard of care in the upfront setting. Further consideration of the implications of interstitial lung disease, a known severe complication of T-DXd, is needed when considering this therapy to treat patients with otherwise curable disease. Table 3 summarizes current studies evaluating T-DXd in patients with early-stage breast cancer.

**Table 3.** T-DXd in the treatment of early-stage breast cancer.

Trial	Design	Setting	Treatment	Outcomes
ADAPT-HER2-IV [NCT05704829]	Phase II	Previously untreated HER2-positive breast cancer with 2 cohorts: (1) low-intermediate risk of recurrence and (2) Intermediate-high risk of recurrence	Neoadjuvant T-DXd monotherapy or chemotherapy plus trastuzumab and pertuzumab (1) for 12 weeks and (2) for 18 weeks	Primary: pCR, dDFS Secondary: iDFS, OS
DESTINY-Breast 05 [NCT04622319]	Phase III	Patients with HER2-positive residual invasive disease after neoadjuvant therapy with either inoperable tumors initially or positive pathologic nodal status	Adjuvant T-DXd vs. T-DM1 following surgery for a year	Primary: iDFS Secondary: DFS, OS
TALENT [NCT04553770]	Phase II Open-label, two-stage	Previously untreated operable breast cancer >2 cm with HER2 low and HR receptor-positive early stage breast cancer	Six cycles of T-DXd with or without anastrozole before surgery	If pCR >10%, evaluation is to be done in a larger trial Prelim results: ORR 63% vs. 75%
DESTINY-Breast 11 [NCT05113251]	Phase III	High-risk HER2-positive breast cancer prior to surgery	Neoadjuvant T-DXd monotherapy or T-DXd followed by THP or ddAC followed by THP	Primary: pCR Secondary: EFS, iDFS, OS

Abbreviations: T-DXd: trastuzumab deruxtecan; T-DM1: adotrastuzumab emtansine; dDFS: distant disease-free survival; iDFS: invasive disease-free survival; DFS: disease-free survival; OS: overall survival; HR: hormone receptor; pCR: pathologic complete response; THP: trastuzumab/pertuzumab/paclitaxel; ddAC: dose-dense doxorubicin/cyclophosphamide; ORR: overall response rate; EFS: event-free survival.

##### Other HER2-Directed ADCs

Trastuzumab duocarmazine (SYD985) is an ADC currently being investigated in HER2-positive and HER2 low breast cancer. The anti-HER2 antibody is bound to a duocarmycin payload, a DNA alkylator. It is also membrane permeable and expected to produce a “bystander effect,“ but has a lower drug-antibody ratio at 2.8:1 compared to 9:1 in T-DXd. Given the manageable safety profile and the anticancer activity noted in phase I clinical trials, it is being further evaluated through larger phase III trials in patients with advanced breast cancer.^73^ In the early-stage setting, a cohort of the ISPY-2 trial is assessing pCR using SYD985 in combination with doxorubicin/cyclophosphamide [NCT01042379].

#### TROP-2 Antibody-Drug Conjugates

TROP-2 is a transmembrane glycoprotein calcium signal transducer, instrumental in intracellular signaling pathways, resulting in proliferation, invasion, and survival of cells. TROP-2 is generally expressed in normal tissues at a low level but overexpressed in certain epithelial tumors.^74^ About 80% of breast cancers have a high-TROP-2 expression, especially HR+/HER2 negative and TNBC.^75-77^

##### Sacituzumab Govitecan

SG, an anti-TROP-2 antibody linked to SN-38 payload and an active metabolite of irinotecan, is the first TROP-2-directed ADC that has been approved for breast cancer treatment. Compared to irinotecan, it can deliver 136 times more SN-38 to cancer cells and induce a bystander effect.^78^ It gained approval following the ASCENT trial, where SG demonstrated improved PFS (5.6 vs. 1.7 months, *P* < .001) and median OS (12.1 vs. 6.7 months, *P* < .001) in patients with metastatic TNBC treated with 2 or more prior regimens.^79^ SG subsequently gained approval for patients with HR+/HER2-negative metastatic breast cancer via the TROPiCS-02 study that demonstrated an improvement in median PFS (5.5 vs. 4 months, *P* = .0003)^80^ and OS (14.4 vs. 11.2 months, *P* = .02).^81^ In addition, a post hoc analysis identified that the benefit of SG was seen in patients irrespective of TROP-2 expression, thus negating the need for TROP-2 testing.^82^

Due to the established role of SG among patients with metastatic breast cancer and an acceptable safety profile, it is being evaluated in the upfront setting for patients with early-stage TNBC. NeoSTAR, a phase II trial, demonstrated a pCR of 30% when patients received neoadjuvant SG.^83^ Given the single-agent efficacy of SG in this study, it will likely be combined with other therapies in future studies to improve pCR rates further.

In the adjuvant setting, SASCIA, a phase III trial [NCT04595565], is currently underway to evaluate the role of adjuvant SG in comparison to capecitabine in patients with residual disease following neoadjuvant therapy for TNBC. More relevant to the current landscape of treatment is the ASCENT-05, an ongoing phase III trial [NCT05633654] that is assessing survival outcomes of adjuvant SG in combination with pembrolizumab versus pembrolizumab with or without capecitabine in patients with residual disease following neoadjuvant therapy. ASPRIA, another ongoing phase II trial of adjuvant SG combined with atezolizumab, is designed to evaluate the efficacy of this combination in patients with residual disease after neoadjuvant therapy and have circulating tumor DNA (ctDNA) in their blood. This strategy could potentially allow for early intervention or escalation of treatment in patients with a higher risk of recurrence based on detectable levels of ctDNA in the blood.

##### Datopotamab deruxtecan

Datopotamab deruxtecan (Dato-DXd) is another ADC directed against TROP-2 with a cleavable tetrapeptide-based linker and a topoisomerase 1 inhibitor payload. Due to its efficacy in the phase I TROPION-PanTumor01 trial in HER2-negative breast cancer,^84^ it is currently being studied in patients with advanced breast cancer via the phase III TROPION-Breast 01 [NCT05104866] and the TROPION-Breast 02 study [NCT05374512]. Notably, preliminary results from the BEGONIA trial evaluating the combination of Dato-DXd with durvalumab as a first-line therapy for patients with advanced metastatic TNBC demonstrated 74% confirmed objective response rates.^85^ Based on this, the TROPION-Breast03 [NCT05629585] is evaluating the role of Dato-DXd in the adjuvant setting either as monotherapy or in combination with durvalumab in comparison to standard of care with capecitabine or pembrolizumab or both, in patients with TNBC and residual disease following neoadjuvant therapy. In addition, I-SPY2, an adaptive randomized trial, is also evaluating Dato-DXd in patients with HER2-negative disease [NCT01042379] in combination with durvalumab in the neoadjuvant setting.

#### Human Epidermal Growth Factor Receptor-3 Antibody-Drug Conjugates

HER3, encoded by the ERBB3 gene, is also a member of the ERBB/HER family but has weaker tyrosine kinase activity. It also dimerizes with other receptors and activates intracellular signaling through the PI3K/AKT and MAPK/ERK pathways.^86^ It has emerged as another potential therapeutic target due to 30%-50% expression in breast cancer.^87^ Patritumab deruxtecan (HER3-DXd) is a monoclonal antibody directed against HER3 bound to the topoisomerase-I inhibitor deruxtecan with a drug-to-antibody ratio of 8. Currently, we only have preliminary data available for this agent in patients with heavily pretreated HER3 expressing metastatic breast cancer, demonstrating an ORR of 30.1% in HR+/HER2-negative breast cancer, 22.6% in HER3 high TNBC and 42.9% in HER3 high HER2-positive breast cancer.^88^ TOT-HER3 [NCT04610528] is a single-arm window-of-opportunity study investigating the response to patritumab deruxtecan in patients with HR+/HER2-negative breast cancer with tumors greater than or equal to 1 cm in size in the early-stage setting.

#### LIV-1 Antibody-Drug Conjugates

LIV-1 is a transmembrane protein with metalloproteinase activity, belonging to a subfamily of ZIP (IRT-like proteins) zinc transporters with heterogenous expression across different normal tissues with high expression in both HR+ breast cancer and TNBC.^89^ Ladiratuzumab vedotin (LV) is an ADC with a humanized monoclonal antibody targeting LIV-1 by a cleavable linker to a cytotoxic payload, monomethyl auristatin E with antitubulin effects. The Phase I SGNLVA-001 trial is investigating LV’s safety and efficacy in patients with pretreated metastatic breast cancer regardless of LIV-1 expression.^90^ An arm of the adaptive I-SPY2 trial also compared LV with an anthracycline-based regimen versus paclitaxel with an anthracycline-based regimen in patients with high-risk stage II or III HER2-negative breast cancer as neoadjuvant therapy. pCR rates were similar; however, rates of peripheral neuropathy were lower among patients in the LV arm.^91^

#### Other Antibody-Drug Conjugates

Several other targets are emerging for the development of ADCs in breast cancer, including Nectin-4, and CEACAM-5, which have also been tested in other solid tumors.^92,93^

Finally, combining ADCs and ICIs as a strategy is gaining momentum particularly in immunogenic subtypes of breast cancer.^94^ Although this is being more extensively evaluated among patients in the metastatic setting, a few trials evaluating this combination in the early stage setting have been summarized in Table 4 and described in the text previously.

**Table 4.** Early-stage breast cancer trials evaluating combination ADCs and ICIs

Trial	Design	Setting	ADC	ICI	Primary outcomes
ASTEFANIA [NCT04873362]	Phase III *N* = 1700	Adjuvant Rx for Stage I-III HER2-positive breast cancer treated with neoadjuvant therapy and residual disease	T-DM1	Atezolizumab	iDFS
ASCENT-05 [NCT05633654]	Phase III *N* = 1514	Adjuvant Rx for TNBC with residual invasive disease after NAT and surgery	SG	Pembrolizumab	iDFS
ASPRIA [NCT04434040]	Phase II *N* = 40	Adjuvant Rx for HER2-negative breast with residual invasive disease after NAT and surgery	SG	Atezolizumab	Rate of undetectable circulating tumor cfDNA
TROPION-Breast 03 [NCT05629585]	Phase III *N* = 1075	Adjuvant Rx for TNBC with residual disease after NAT and surgery	Dato-DXd	Durvalumab	iDFS
I-SPY2 [NCT01042379]	Phase II adaptive *N* = 5000	NAT for HER2-negative disease × 4 cycles	Dato-DXd	Durvalumab	pCR

Abbreviations: Rx: treatment; HER2: human epidermal growth factor receptor-2; iDFS: invasive disease-free survival; T-DM1: adotrastuzumab emtansine; SG: sacituzumab govitecan; NAT: neoadjuvant therapy; cfDNA: cell-free DNA; Dato-DXd: datopotomab deruxtecan; pCR: pathologic complete response.

#### Future directions of ADCs

Overall, ADCs are expanding the therapeutic options for patients with breast cancer. They are ultimately transforming chemotherapy into a targeted means of treatment with their ability to deliver cytotoxic drugs specifically to tumor cells. The prospect of its efficacy in early disease settings is promising, given its ability to offer favorable responses even in heavily pretreated patients. However, this needs to be carefully weighed against the adverse effects of these medications. With a plethora of new ADC options, questions arise regarding the optimal timing and sequencing of these drugs in patients with overlapping candidacies. One aspect of ADCs that could help with therapeutic sequencing is understanding resistance mechanisms that could occur with the antibody or the payload.^95^ Moreover, while some clinical trials of ADCs include only patients whose tumors express a specific target antigen, others, like SG, were conducted with no biomarker selection. Future therapies, including ADCs directed against new targets, may make biomarker selection necessary for clinical decision-making. This approach requires not only investigation of predictive biomarkers but also validated assays to identify what level of expression of an antigen is considered positive. In addition, this expression can change over the course of a patient’s disease or vary due to tumor heterogeneity.

## Conclusion

In summary, while ICIs and ADCs represent a promising shift in the paradigm of breast cancer treatment, it is of utmost importance to tailor them appropriately to the patients who would most benefit from them. This requires a sustained commitment to biomarker identification and efforts to optimize toxicities, including immune-related and financial, to achieve an ideal implementation strategy.

## Data Availability

No new data were generated or analyzed during the preparation of this manuscript.
