# Correction to: The Evolving Landscape of Immune Checkpoint Inhibitors and Antibody Drug Conjugates in the Treatment of Early-Stage Breast Cancer

**DOI:** 10.1093/oncolo/oyad330

**Published:** 2023-12-13

This is a correction to Prarthna V. Bhardwaj, Yara G. Abdou, The Evolving Landscape of Immune Checkpoint Inhibitors and Antibody Drug Conjugates in the Treatment of Early-Stage Breast Cancer, *The Oncologist*, Volume 28, Issue 10, October 2023, Pages 832–844, https://doi.org/10.1093/oncolo/oyad233

On page 833, in the first paragraph of the Checkpoint Inhibitors in Triple-Negative Breast Cancer section, the pathologic complete response shown for the KEYNOTE-522 study was incorrect “64.8% vs. 51.2%” should have been “63% vs. 55.6%”.

Reference 16 is cited at the end of this sentence, but reference 16 published incorrectly in the reference list. The reference list showed a reference to the original study (Schmid P, Cortes J, Pusztai L, et al. Pembrolizumab for early triple-negative breast cancer. *N Engl J Med.* 2020;382(9):810-821. https://doi.org/10.1056/NEJMoa1910549), but should have shown the following: Schmid P, Cortes J, Dent R, et al. Event-free survival with pembrolizumab in early triple-negative breast cancer. *N Engl J Med.* 2022;386:556-567. https://doi.org/10.1056/NEJMoa2112651.

The last sentence on page 833 is also in error. Regarding availability of atezolizumab for patients with early-stage triple-negative breast cancer, “including the United States” should be deleted. The sentence should read as follows: However, it remains an option for patients in the rest of the world, subject to availability.”

The following errors affect Table 1 on page 834:

The references cited in the column headings are in error, and should appear as follows:

KEYNOTE 522 – Reference 16

NeoPACT – Reference 28

Impassion031 – Reference 19

NeoTRIP – No change

GeparNuevo – Reference 17

I-SPY – Reference 43

In the KEYNOTE 522 column, the number of patients should be 1174 (instead of 602); the pCR (ICI vs. placebo) should show 63% vs 55.6% (instead of 64.8% vs. 51.2%); and the estimated treatment difference should show 7.5% (95% CI: 1.6%, 13.4%) (instead of 13.6 percentage points; 95% CI, 5.4-21.8).

